# Enhancing the Stability and Bioaccessibility of Tree Peony Seed Oil Using Layer-by-Layer Self-Assembling Bilayer Emulsions

**DOI:** 10.3390/antiox12051128

**Published:** 2023-05-20

**Authors:** Wen-Sen He, Qingzhi Wang, Zhishuo Li, Jie Li, Liying Zhao, Junjie Li, Chen Tan, Fayong Gong

**Affiliations:** 1 School of Food and Biological Engineering, Jiangsu University, 301 Xuefu Road, Zhenjiang 212013, China; 2 Beijing Engineering and Technology Research Center of Food Additives, Beijing Technology & Business University (BTBU), Beijing 100048, China; 3 Panxi Crops Research and Utilization Key Laboratory of Sichuan Province, Xichang University, Xichang 615013, China

**Keywords:** α-linolenic acid, tree peony seed oil, bioavailability, n-3 polyunsaturated fatty acid, stability, bilayer emulsion, encapsulation, delivery

## Abstract

Tree peony seed oil (TPSO) is an important plant source of n-3 polyunsaturated fatty acid (α-linolenic acid, ALA > 40%) that is receiving increasing attention for its excellent antioxidant and other activities. However, it has poor stability and bioavailability. In this study, a bilayer emulsion of TPSO was successfully prepared using a layer-by-layer self-assembly technique. Among the proteins and polysaccharides examined, whey protein isolate (WPI) and sodium alginate (SA) were found to be the most suitable wall materials. The prepared bilayer emulsion contained 5% TPSO, 0.45% whey protein isolate (WPI) and 0.5% sodium alginate (SA) under selected conditions and its zeta potential, droplet size, and polydispersity index were −31 mV, 1291 nm, and 27%, respectively. The loading capacity and encapsulation efficiency for TPSO were up to 84% and 90.2%, respectively. It was noteworthy that the bilayer emulsion showed significantly enhanced oxidative stability (peroxide value, thiobarbituric acid reactive substances content) compared to the monolayer emulsion, which was accompanied by a more ordered spatial structure caused by the electrostatic interaction of the WPI with the SA. This bilayer emulsion also exhibited markedly improved environmental stability (pH, metal ion), rheological properties, and physical stability during storage. Furthermore, the bilayer emulsion was more easily digested and absorbed, and had higher fatty acid release rate and ALA bioaccessibility than TPSO alone and the physical mixtures. These results suggest that bilayer emulsion containing WPI and SA is an effective TPSO encapsulation system and has significant potential for future functional food development.

## 1. Introduction

Tree peony seed oil (TPSO) is a woody oil extracted from the seeds of Fengdan (*Paeonia ostii* T. *Hong et J.X. Zhang*) and Ziban (*Paeonia rockii* T. *Hong et J. J. Li*) tree peony. TPSO has a high unsaturated fatty acid content (>90%), contains up to 40% α-linolenic acid, and is an important source of n-3 polyunsaturated fatty acids (PUFA) [1]. TPSO also contains phytosterols [2], squalene [3], tocopherols [4], and other active ingredients. Numerous studies have proposed that TPSO exerts a wide range of beneficial health effects, including hypolipidemic, hepatoprotective, and antioxidative effects [5]. Besides, TPSO can effectively alleviate hepatic oxidative damage of rats induced by high-fat diet [6]. Therefore, TPSO has a significant potential for development as a health food, with the Chinese Ministry of Health approving it as a new resource food in 2011. However, TPSO is rich in PUFAs that contain many double bonds, which are extremely sensitive to oxygen, light, and heat and are easily oxidized and rancidified [7]. In addition, the slow intestinal digestion and hydrolysis of linolenic acid-rich oils and their low oral bioavailability are not conducive to high levels of bioactivity [8]. It has been reported that less than 2% of omega-3 fatty acid alpha-linolenic acid (ALA) is converted into the bioactive eicosapentaenoic and docosahexaenoic acids in the body [9].

To overcome these problems, an effective delivery strategy for TPSO is required. Although these conventional emulsions, known as monolayer emulsions, have previously proven to be effective carriers for the delivery of functional oils and fats [10], they only form a monolayer interface at the oil–water interface in the presence of emulsifiers alone and tend to be unstable during storage due to flocculation, coagulation, and gravitational separation [11]. In addition, the main emulsifiers currently used for the preparation of TPSO emulsions, Tween 80 (polyoxyethylene sorbitan monooleate) and Span 80 (sorbitan monooleate), are chemically synthesized and may be potentially hazardous [12,13,14]. Electrostatic layer-by-layer self-assembly is an emerging technology that uses electrostatic adsorption between cationic and anionic polyelectrolytes to deposit alternating layers on the surfaces of charged template materials to form multilayer interfacial films [15]. Compared with conventional monolayer emulsion, bilayer or multilayer emulsions prepared using this technology have greater stability and longer shelf lives due to the thick and dense interfacial layers, high electrostatic repulsion, and good interfacial properties. The formation of thick interfacial layers around oil droplets by electrostatic layer self-assembly can be used as a strategy to protect lipolytic functional factors from degradation and improve their bioaccessibility [16].

**Figure 1:**
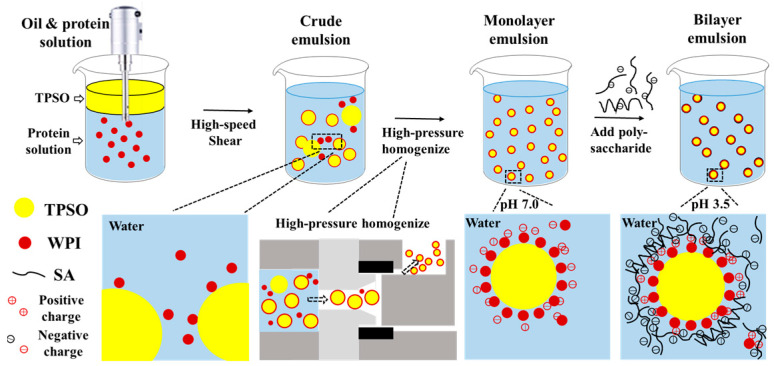
Schematic representation of preparing TPSO bilayer emulsion constructed by layer-by-layer self-assembly technology.

In this study, a bilayer emulsion of TPSO was fabricated using electrostatic layer-by-layer self-assembly technique (Figure 1). The rheological properties and circular dichroism of mono- and bilayer emulsions were explored. In addition, the oxidative stability, physical stability, and environmental stability and in vitro gastrointestinal digestibility (fatty acid release, bioaccessibility) of TPSO bilayer emulsions were studied.

## 2. Materials and Methods

### 2.1. Materials

Food-grade TPSO was supplied by Heze Ruipu Peony Industry Technology Development Co., Ltd. (Heze, Shandong, China). The composition of major fatty acids and beneficial components in TPSO was shown in Table S1. Whey protein isolate (WPI, food grade) was purchased from Zhengzhou Weifeng Biotechnology Co., Ltd. (Zhengzhou, Henan, China). Soybean protein isolate (SPI, biological reagent grade), mucin (porcine stomach, biological reagent grade), pepsin (porcine gastric mucosa, 30,000 U/g), bile salt (pig, cholic acid content ≥ 60%), and pancreatin (pancreatic lipase activity ≥ 4000 U/g) were supplied by Yuanye Biological Technology Co., Ltd. (Shanghai, China). Nile red was purchased from Shanghai Macklinn Biochemical Technology Co., Ltd. (Shanghai, China). Gelatin (GE, chemical pure grade), sodium alginate (SA, chemical pure grade), gum arabic (GA, biological reagent grade), sodium carboxymethyl cellulose (CMC-Na, chemical pure grade), sodium benzoate, and other reagents of analytical grade were obtained from Sinopharm Chemical Reagents Co., Ltd. (Shanghai, China).

### 2.2. Preparation of Polysaccharide and Protein Solutions and Zeta Potential Determination

WPI solutions of various concentrations were prepared by dissolving WPI in a 10 mM, pH 7 phosphate buffer solution (PBS) with 0.02% sodium benzoate as a preservative, mixing thoroughly, and centrifuging at 5000 rpm for 10 min. The same method was used for the preparation of GE, SPI, GA, SA, and CMC-Na solutions. All solutions were diluted to 0.01% with PBS, and then the pH was adjusted with 1 M HCl or 1 M NaOH to measure their zeta potential at different pH values. A 700 µL sample was placed in an omega sample tank and its zeta potential was measured with a Litesizer 500 particle size analyzer at 25 °C with a solvent refractive index of 1.33; the approximation and Henry factor is Smoluchowski and 1.5 under the maximal voltage of 200 V, separately.

### 2.3. Preparation of Monolayer Emulsion

TPSO (10 g) was added to 90 g of protein solution and sheared at 12,000 rpm for 3 min to obtain a crude emulsion. Subsequently, the crude emulsion was homogenized at 400 bar and repeated 3 times to obtain a monolayer emulsion (Figure 1).

### 2.4. Preparation of Bilayer Emulsion

Bilayer emulsions were prepared by adding 100 g polysaccharide solution (0.25–1.5% GA, SA, or CMC-Na) to 100 g monolayer emulsion prepared in Section 2.3, stirring at 300 rpm for 20 min, adjusting the pH to 3.5, and mechanically shearing at 8000 rpm for 2 min (Figure 1).

### 2.5. Measurement of Emulsion Activity Index and Emulsion Stability Index

The emulsion activity index (EAI) and emulsion stability index (ESI) were determined as according to a previous method with slight modification [17]. Briefly, 0.5% (*w*/*v*) protein solution was mixed with TPSO at a volume ratio of 9:1 and homogenized at 8000 rpm for 2 min. The resulting crude emulsion was diluted 200 times with 0.1% sodium dodecyl sulfate solution (SDS) and its absorbance values at 0 and 30 min were measured.

(1) $$EAI(\frac{m^{2}}{g})=\frac{2\times 2.303\times A_{0}\times N}{10000\times C\times L\times \psi },$$

(2) $$ESI(min)=\frac{t\times A_{0}}{A_{0}-A_{30}},$$

where *A*_0_ and *A*_30_ are the absorbance value at 0 and 30 min, N is the dilution ratio (200), *C* is the protein concentration (g/mL), *L* is the diameter of the colorimetric dish optical path (1 cm), *ψ* is the volume fraction of oil in lotion (1/10), and *t* is 30 min.

### 2.6. Determination of Emulsion Zeta Potential, Droplet Size, and PDI

The monolayer and bilayer emulsions were diluted 100- and 500-fold with PBS (10 mM) at pH 7 and 3.5. A total of 1 mL of the diluted emulsion was placed in a standard disposable cuvette and its droplet size and PDI were measured on a Litesizer 500 particle size analyzer using dynamic light scattering in backscattering mode at a 175 angle. All measurements were performed at 25 °C with a refractive index of 1.45 and 1.33 for samples and solvent, respectively. The zeta potential was measured as described in Section 2.2.

### 2.7. Measurement of Encapsulation Efficiency

Encapsulation efficiency was determined according to previous methods with slight modifications [18]. Specifically, 1.5 g of lyophilized sample of monolayer or bilayer emulsions was mixed with 30 mL of petroleum ether for 3 min and then centrifuged at 5000 rpm for 10 min to obtain a petroleum ether layer. The surface oil weight m_0_ was determined after drying. The initial amount of TPSO added during emulsion preparation was defined as the total oil weight m_1_. The encapsulation efficiency was then calculated using the following equation:

Encapsulation efficiency (%) = (1 − m_0_/m_1_) × 100 (3)

### 2.8. Microstructure Observation

The monolayer and bilayer emulsions were diluted to 1% of the TPSO content with pH 7 and 3.5 PBS, and 10 μL of each emulsion was placed on a slide and observed using a BX53-P microscope (Olympus, Tokyo, Japan). Nile Red ethanol solution (40 μL of a 1 mg/mL solution) was added to 1 mL of each diluted emulsion and mixed well [19]. A 10-μL stained sample was placed on a slide and observed using a CTR4000B inverted fluorescence microscope.

### 2.9. Rheological Properties

The rheological properties of the mono- and bilayer emulsions were determined using a DHR-1 rheometer. Apparent viscosity was measured by steady-state flow with a plate diameter of 40 mm and a shear rate of 0.1–100 s^−1^. All dynamic tests were performed within the linear viscoelastic region. The moduli of elasticity (G′) and loss (G″) were determined with a plate rotation frequency of 0.1–10 rad/s and 1% strain [20,21].

### 2.10. Determination of Circular Dichroism

The circular dichroism of the mono- and bilayer emulsions was determined using a J-815 circular dichroism spectrometer at a wavelength of 190–250 nm, a bandwidth of 1 nm, and an interval of 0.5 nm/s. PBS and SA solutions were used as controls for determining WPI intensity in the mono- and bilayer emulsions, respectively [22].

### 2.11. Physical Stability and Oxidative Stability during Storage

The mono- and bilayer emulsions were stored at 4 °C and 25 °C for 30 days, and the droplet size, zeta potential, PDI, cream index (CI), peroxide value (POV), and thiobarbituric acid reactive substances (TBARS) content were measured periodically. The microstructure and appearance of each emulsion were also observed. Droplet size, zeta potential, and PDI were determined for each emulsion, as described in Section 2.6.

CI was determined using the following formula [23]:

CI (%) = Hc/Ht × 100,(4)

where Hc is the creaming height (cm) and Ht is the total emulsion height (cm).

The POV and TBARS content of the samples were determined using the methods of Gudipati et al. (2010) [24] and Chen et al. (2016) [25], respectively. In brief, the hydroperoxides in the emulsion were extracted with isooctane/isopropanol (3:1, *v*/*v*) solution, and the color development reaction was carried out by adding methanol/n-butanol (2:1, *v*/*v*), ammonium thiocyanate and ferrous chloride solution. The absorbance value at 510 nm was measured, and the lipid hydroperoxide concentration (μmol/L emulsion) was calculated from the standard curve made of hydrogen peroxide isopropylbenzene, and the POV value was expressed as mmol/kg oil per unit mass of oil. Similarly, the emulsion was reacted with TBA reagent in a boiling water bath for 15 min and the supernatant was collected by centrifugation to determine its absorbance value at 532 nm. The TBARS concentration (μmol/L emulsion) was determined from the standard curve of 1,1,3,3-tetraethoxypropane and converted to in terms of oil (mmol/kg oil).

### 2.12. Effect of pH on the Stability of Emulsion

The newly prepared monolayer and bilayer emulsions were adjusted to pH 2~8 with 1 M HCl or NaOH at 300 rpm. After 1 h at 4 °C, the zeta potential, droplet size, and PDI were measured as described in Section 2.6 and the appearance of emulsion was also recorded by taking pictures.

### 2.13. Effect of Metal Ion on the Stability of Emulsion

The newly prepared monolayer and bilayer emulsions was diluted 100 times with 0~0.5 M NaCl or CaCl_2_ at 300 rpm. After 1 h at 4 °C, the zeta potential, droplet size, and PDI were measured as described in Section 2.6 and the appearance of the emulsion was also recorded by taking pictures.

### 2.14. In Vitro Gastrointestinal Digestion

The in vitro digestion of pure TPSO, physical mixtures (PM), monolayer emulsions (ME), and bilayer emulsions (BE) in the gastrointestinal tract was determined according to a previous method with slight modifications [26,27]. The PM sample contained the same amount of TPSO and WPI as the monolayer emulsion, while the pure TPSO sample did not contain WPI. Both pure TPSO and PM samples were not homogenized. Prior to digestion, the samples were diluted with PBS to a TPSO content of 4 wt%. Digestion was performed at 37 °C and 100 rpm to simulate the human digestive tract.

*Oral digestion*. Simulated salivary fluid was mixed with an equal volume of the sample containing 4 wt% TPSO and digested at pH 6.8 for 2 min [28]. The samples were sequentially passed through an entire simulated oral–gastric–intestinal digestive tract, but digestion of the samples following oral digestion was not examined as oil is predominantly digested in the stomach and intestine.

*Gastric digestion*. Following oral digestion, the samples were mixed with an equal volume of simulated gastric fluid consisting of 3.2 mg/mL pepsin, 0.7% hydrochloric acid, and 2 mg/mL sodium chloride, and the pH was adjusted to 2.0 with 1 M hydrochloric acid. The samples were digested for 2 h.

*Intestinal digestion*. Following gastric digestion, 30 mL of the sample was adjusted to pH 7, and 4 mL of bile salt solution (46.87 mg/mL), 1.0 mL of CaCl_2_ solution (187.5 mM), 1.0 mL of NaCl solution (3.75 M), and 1.5 mL of 40 mg/mL of trypsin solution were added and mixed for 3 h of digestion. Free fatty acid (FFA) release was calculated using the pH-stat titration method per the formula [29]:

FFA (%) = (V_NaOH_ × C_NaOH_ × M_lipid_ × 100)/(W_lipid_ × 2), (5)

where V_NaOH_ and C_NaOH_ are the volume and concentration, respectively, of the NaOH solution used during the simulated small intestine digestion, and M_lipid_ and W_lipid_ are the average molecular weight and total weight, respectively, of the TPSO added to the emulsion at the beginning of digestion.

The micelle layer (middle) of intestinal digest was obtained by centrifugation at 10,000× *g* for 40 min, as previously described [30]. The micelles were first extracted with *n*-hexane, then derivatized with boron trifluoride and subjected to gas chromatographic analysis performed on a 2010 Plus gas chromatograph using heptadecanoic acid as an internal standard [31,32]. The bioaccessibility of ALA was calculated according to the following formula [30]:

ALA bioaccessibility (%) = m_1_ × 100/m_2_, (6)

where m_1_ represents the mass of ALA in the micelle (mg), and m_2_ represents the initial mass of ALA before digestion (mg).

### 2.15. Statistical Analyses

All experiments were performed at least three times, and the results are expressed as the mean ± standard deviation. The results of the storage and digestion experiments were analyzed by two-way analysis of variance (ANOVA) using Prism 9.3.0 software (GraphPad, San Diego, CA, USA). All other data were analyzed by one-way ANOVA and the Student–Newman–Keuls post-hoc test using SPSS 26.0 (IBM, Armonk, NY, USA), with a significance threshold of *p* < 0.05. Data marked with different letters were significantly different.

## 3. Results and Discussion

### 3.1. Determination of Monolayer Emulsifier

**Figure 2:**
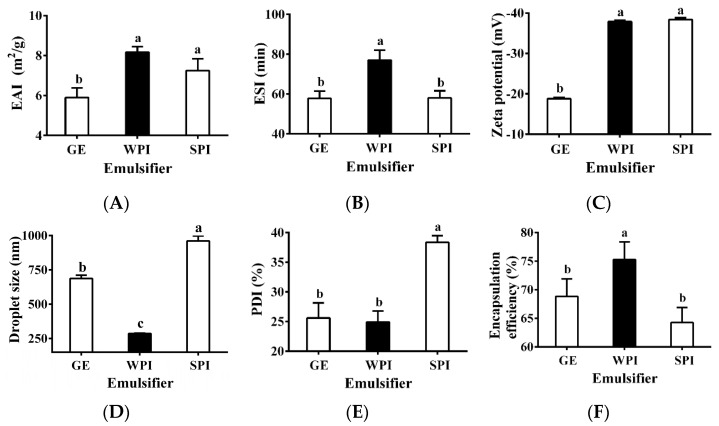

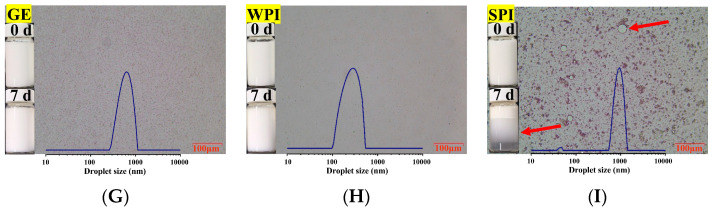
Physical properties of monolayer emulsion in three groups. (**A**) Emulsion activity index (m^2^/g). (**B**) Emulsion stability index (min). (**C**) Zeta potential (mV). (**D**) Droplet size (nm). (**E**) PDI (%). (**F**) Encapsulation efficiency (%). (**G**–**I**) The appearance and microstructure of emulsion in GE, WPI, and SPI group, individually. Red arrows represent the appearance of small oil droplets and phase separation in SPI-stabilized monolayer emulsions after seven days of storage. GE: gelatin group (*n* = 3); WPI: whey protein isolate group (*n* = 3); SPI: soybean protein isolate group (*n* = 3). One-way ANOVA followed by LSD test was used for statistical significance. Data marked with different superscript letters (a, b, c) differ significantly at *p* < 0.05.

Three common proteins, GE, WPI, and SPI, were selected as potential emulsifiers (i.e., inner wall material) to produce TPSO monolayer emulsions. The emulsion activity index (EAI) and emulsion stability index (ESI) are important indicators of the emulsifying properties of a specific protein [33], and WPI had the highest EAI and ESI (Figure 2A,B). Zeta potential is another important indicator used to characterize the stability of colloidal dispersion systems. When the absolute zeta potential is >30 mV, an emulsion can be considered stable due to sufficient electrostatic repulsive force [34,35]. The zeta potentials of TPSO monolayer emulsions prepared with WPI and SPI were comparable, close to −40 mV, while that of the GE monolayer emulsion was significantly lower (Figure 2C), indicating that both WPI and SPI confer extremely strong resistance to oil droplet aggregation [36]. Droplet size and PDI have important influence on the stability of emulsions [37]. Among the three monolayer emulsions, the WPI emulsion had the smallest droplet size (286.6 nm), the lowest PDI (24.9%), and the greatest encapsulation efficiency (Figure 2D–F). The appearances of the three monolayer emulsions were not significantly different initially (Figure 2G–I). However, after seven days of storage, small oil droplets could be observed in the SPI-stabilized monolayer emulsion, and significant phase separation and slight oil spillage were seen (Figure 2I). This indicated that SPI was less capable of emulsifying and stabilizing TPSO than WPI. Therefore, WPI was selected as the most suitable emulsifier for the monolayer emulsification of TPSO.

### 3.2. Determination of WPI Concentration

The physical properties, microstructure, and morphology of TPSO monolayer emulsions prepared with different concentrations of WPI were investigated (Figure S1). A TPSO monolayer emulsion stabilized with 0.5% WPI had relatively large droplets (304.9 nm), reduced encapsulation efficiency (60.7%), and lower zeta potential (−36 mV) (Figure S1A,B,D). However, no significant oil droplet aggregation was seen for the 0.5% WPI emulsion (Figure S1E). For this emulsion, the relatively low WPI concentration may not have been sufficient to adequately emulsify and stabilize the TPSO, resulting in a large droplet size and low encapsulation rate. Sun et al. (2019) reported a similar finding when using a low concentration of linseed gum to stabilize olive oil [38]. These indexes significantly improved with increasing WPI concentration (Figure S1A,B,D). For a 1% WPI emulsion, the droplet size decreased to 287.2 nm, the encapsulation rate increased to 74%, and the zeta potential approached −38 mV (Figure S1A,B,D), indicating that an increase in WPI concentration could stabilize the TPSO monolayer emulsion (Figure S1F). However, further increasing the WPI concentration (1.5–2.5%) did not result in a significant increase in the zeta potential or encapsulation rate, while the droplet size increased. Moreover, these high-concentration monolayer emulsions were stratified after 7 days of storage (Figure S1G–I). This may be due to the saturation of the TPSO with the emulsifier [39]. The PDI of the TPSO monolayer emulsion was found to be independent of the WPI concentration (Figure S1C). When preparing a bilayer emulsion, the WPI concentration should be minimized to prevent excess WPI from interacting with oppositely charged polyelectrolytes in the bilayer emulsion [40]. Therefore, a WPI concentration of 1% was chosen for preparing the TPSO monolayer emulsion, resulting in an emulsion containing 10% TPSO and 0.9% WPI.

### 3.3. Selection of Polysaccharide and Determination of pH

**Figure 3:**
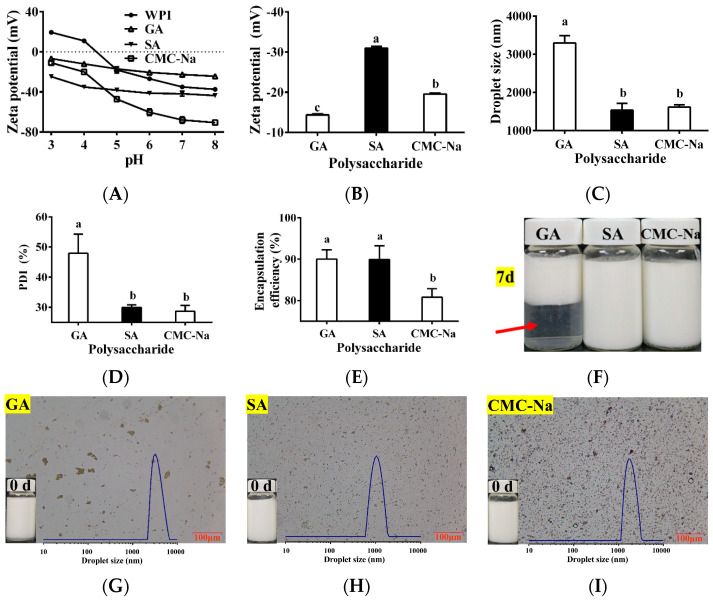
Physical properties of bilayer emulsion in three groups (*n* = 3). (**A**) Zeta potential of WPI, GA, SA, and CMC-Na at different pH (mV). (**B**) Zeta potential (mV). (**C**) Droplet size (nm). (**D**) PDI (%). (**E**) Encapsulation efficiency (%). (**F**) The appearance of bilayer emulsion in three groups after seven days. Red arrows represent delamination of the GA prepared bilayer emulsion after 7 days of storage. (**G**–**I**) The appearance and microstructure of the emulsion in the GE, SA, and CMC-Na group. One-way ANOVA followed by LSD test was used for statistical significance. Data marked with different superscript letters (a, b, c) differ significantly at *p* < 0.05.

Three common polysaccharides (GA, SA, and CMC-Na) were selected as potential outer wall materials for the TPSO bilayer emulsion. The zeta potential of bilayer emulsions composed of WPI and GA, SA, or CMC-Na are shown in Figure 3A. GA, SA, and CMC-Na are anionic polysaccharides that are negatively charged at pH 3–8 [41,42,43]. WPI is an amphoteric electrolyte with an isoelectric point of pH 4.3, below which it is positively charged and above which it is negatively charged. Therefore, WPI can only be electrostatically attracted to the anionic polysaccharides below its isoelectric point (pH < 4.3). The greater the positive charge of the protein and the greater the negative charge of the polysaccharide, the stronger the electrostatic attraction between the two and the stronger the interfacial layer formed is, and vice versa. Additionally, the higher the absolute value of the electrostatic charge remaining after charge neutralization between the protein and polysaccharide, the more stable the bilayer emulsion. At pH 3–4, SA had the largest absolute negative charge, followed by CMC-Na and then GA (Figure 3A), making SA the most suitable outer wall material for the preparation of the TPSO bilayer emulsion in theory. Different TPSO bilayer emulsions were subsequently prepared at pH 3.5 using either GA, SA, or CMC-Na as the outer wall material, and their physical properties, microstructures, and morphologies are shown in Figure 3B–I. When SA and CMC-Na were used as the outer wall materials, the droplet size and PDI of the resulting TPSO bilayer emulsions were small (Figure 3C,D). However, the encapsulation rate for CMC-Na was also low, and significant oil droplet was observed (Figure 3E,I). However, the absolute zeta potential of the bilayer emulsion prepared with GA was much smaller than that containing SA (Figure 3B). The bilayer emulsion prepared with GA had a maximum droplet size of 3295 nm, a PDI of 48.0 ± 6.3%, and a non-uniform distribution of emulsion droplets, with significant delamination after 7 days of storage (Figure 3C,D,F,G). Therefore, SA was selected as the most suitable outer wall material.

### 3.4. Determination of SA Concentration

The physical properties and microscopic morphologies of TPSO bilayer emulsions produced using different concentrations of SA were then examined (Figure S2). Among the different concentrations, the 0.25% SA-stabilized TPSO bilayer emulsion had the largest droplet size and PDI, the smallest zeta potential, and the lowest TPSO encapsulation rate (<80%) (Figure S2A–D). This concentration of SA was not sufficient to completely cover the droplet surface, and one SA could become adsorbed to multiple droplets, leading to bridging flocculation and increased droplet size [44]. As a result, the drops accumulated and phase separation occurred after only one week of storage (Figure S2E,L). With increasing concentrations of SA, the positively charged WPI layer was gradually neutralized, resulting in a negatively charged bilayer emulsion. The absolute value of this negative charge increased correspondingly with increasing SA concentrations (Figure S2A). The droplet size and PDI decreased with increasing SA concentration (Figure S2B,C). When 1% SA was used, the zeta potential of the resulting bilayer emulsion was maximized (−31 mV) and the droplet size (1291 nm) and PDI (27%) were minimized (Figure S2A–C), suggesting that the SA molecules fully coated the WPI molecules encapsulating the oil droplets at this concentration [45]. Moreover, this 1% SA-stabilized bilayer emulsion had the highest TPSO encapsulation rate (Figure S2D). After being kept fresh or stored for 7 days, the 1% SA-stabilized TPSO bilayer emulsion was stable and showed uniform dispersion (Figure S2H,L), indicating that 1% SA was sufficient for the formation of a stable bilayer emulsion. A solid negatively charged bilayer interfacial layer was formed due to the 1% SA electrostatically adsorbing to the WPI in the monolayer emulsion, forming a solid negatively charged bilayer interfacial layer. The TPSO oil droplets within these interfacial shells were less likely to aggregate due to strong electrostatic repulsion, forming a stable dispersion system. The zeta potential, droplet size, and PDI of the bilayer emulsion prepared with 1% WPI and 1% SA were −31 mV, 1291 nm, and 27%, respectively, with an encapsulation efficiency of 90.2%. Under the selected conditions, the prepared bilayer emulsion contained 5% TPSO, 0.45% WPI, and 0.5% SA.

### 3.5. Rheological Properties

**Figure 4:**
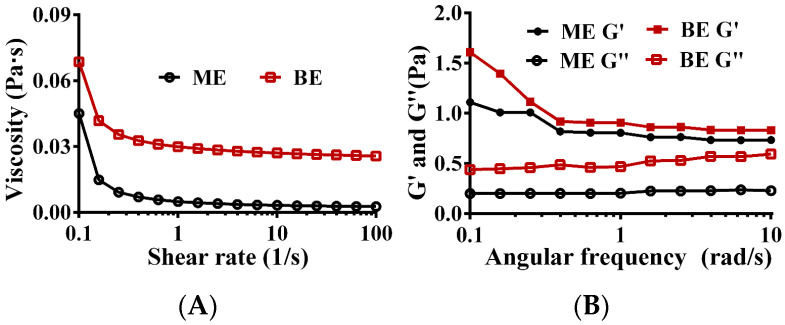
The rheological properties of emulsion. (**A**) The relationship between shear rate and emulsion viscosity. (**B**) The relationship between angular frequency and energy storage modulus and loss modulus of emulsion. ME: monolayer emulsion; BE: bilayer emulsion; ME G′: the storage modulus of monolayer emulsion; ME G″: the loss modulus of monolayer emulsion; BE G′: the storage modulus of bilayer emulsion; BE G″: the loss modulus of bilayer emulsion.

Rheological properties are important indicators of the stability of emulsions. Due to its high viscosity, the emulsion neither floated nor settled and exhibited good physical stability. As shown in Figure 4A, with increasing shear rates, the viscosity of both the mono- and bilayer emulsions decreased and exhibited shear thinning, suggesting that both are non-Newtonian fluids. Regardless of the shear rate (0.1–100 s^−1^), the viscosity of the bilayer emulsion was greater than that of the monolayer emulsion, indicating superior stability of the bilayer emulsion. This is likely due to the electrostatic interactions between the WPI and SA molecules, which confer stability upon the oil–water interfacial layer. Consistently, a similar phenomenon has been reported for a multilayer emulsion of bitter gourd seed oil using WPI, apple pectin, and chitosan oligosaccharide [46].

The energy storage modulus (G′), also known as the elastic modulus, and the loss modulus (G″), reflect the elastic and viscous behaviors, respectively, of an emulsion network structure. The lower the G′, the weaker the interaction forces between the emulsion droplets and the easier deformation is when subjected to external forces. The G′s of both the mono- and bilayer emulsions were always greater than the G″s, and they did not intersect (Figure 4B), indicating that both emulsions formed a gel network structure dominated by elasticity. Regardless of angular frequency, the G′s of the bilayer emulsions were always higher than those of the monolayer emulsion (Figure 4B), indicating that the monolayer emulsion viscoelasticity was significantly enhanced by the addition of SA. The addition of SA at the oil–water interface resulted in increased thickness of the interfacial film and reduced interdroplet space, making the bilayer emulsion more resistant to deformation. Additionally, any unadsorbed SA in the continuous phase would increase the emulsion viscosity and contribute to an enhanced emulsion gel structure.

### 3.6. Circular Dichroism

WPI is a protein isolated from milk, mainly including β-lactoglobulin, α-lactalbumin, bovine serum albumin, etc. The circular dichroism of WPI and the mono- and bilayer emulsions are shown in Table 1. When incorporated in a monolayer emulsion, the α-helix content of WPI was reduced by 52.1%, and the β-folding and β-turning angles were correspondingly increased by 67.0% and 7.2%, respectively. In a peptide chain, α-helices are mainly stabilized by the hydrogen bonds between amino hydrogen (NH-) and carbonyl oxygen (CO-). The reduced α-helix content of WPI in a monolayer emulsion may result from the disruption of hydrogen bonds during the high-pressure homogenization phase of its preparation. Lee et al. (2008) reported that protein bilayer structures are associated with homogenization. Increasing homogenization pressures were reported to result in decreased whey protein α-helix content in a soybean oil-lactalbumin emulsion, with a corresponding increase in β-folding [47]. In addition, high-pressure homogenization generates heat, which can also alter protein secondary structures. Han et al. (2015) investigated the effects of temperature on myosin secondary structures and found that the α-helix content decreased and β-folding increased within a certain temperature range [48]. Compared with the monolayer emulsion, the α-helix and β-turn content of WPI in the bilayer emulsion increased by 25.6% and 16.2%, respectively. These changes were accompanied by a corresponding 12.3% decrease in random curl structures, suggesting that the secondary structure of WPI became more ordered after forming electrostatic interactions with SA. However, the mono- and bilayer emulsions were prepared at different pH values, potentially influencing the secondary protein structure. For example, Sun et al. (2018) reported an increase in the α-helix content and a decrease in random curl content in particulate whey protein prepared at pH 3.5 compared to pH 6.5 and 7.5 [49].

### 3.7. Physical and Oxidative Stability

**Figure 5:**
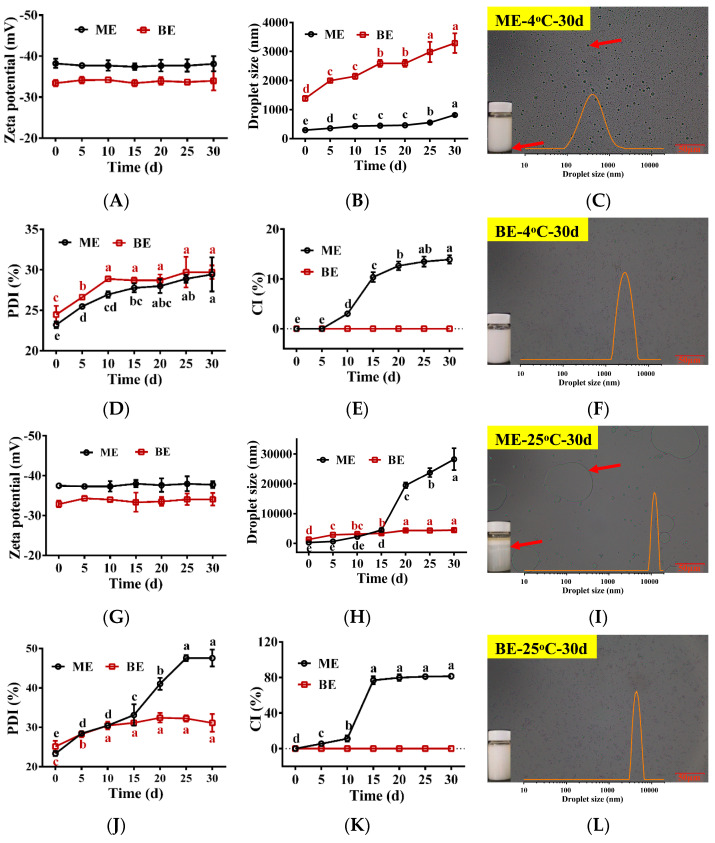
The physical stability of emulsions during storage (*n* = 3). (**A**–**F**) Zeta potential (mV), droplet size (nm), the appearance and microstructure of monolayer emulsion after 30 days of storage, PDI (%), CI (%) and the appearance and microstructure of bilayer emulsion after 30 days of storage at 4 °C, separately. (**G**–**L**) Zeta potential (mV), droplet size (nm), the appearance and microstructure of monolayer emulsion after 30 days of storage, PDI (%), CI (%) and the appearance and microstructure of bilayer emulsion after 30 days of storage at 25 °C, separately. Red arrows represent delamination and aggregation of oil droplets in the emulsion. ME: monolayer emulsion; BE: bilayer emulsion. One-way ANOVA followed by LSD test was used for statistical significance. Data marked with different superscript letters (a, b, c, d, e) differ significantly at *p* < 0.05.

Next, we investigated the changes seen in the physical properties of TPSO mono- and bilayer emulsions stored at 4 °C and 25 °C (Figure 5). The droplet sizes and PDIs of mono- and bilayer emulsions increased with storage time, while the zeta potentials did not change significantly (Figure 5A,B,D,G,H,J). Specifically, the droplet sizes of mono- and bilayer emulsions were 2.9- and 2.3-fold larger when kept at 4 °C for 30 days, respectively, with the droplet size of the bilayer emulsion always remaining higher than that of the monolayer emulsion. At 25 °C, the droplet size and PDI of the monolayer emulsion were smaller than those of the bilayer emulsion for the first 10 days. However, from 15 days onwards the opposite relationship was observed. By 30 days of storage, the droplet size of the monolayer emulsion had reached 28,283 nm, a 99.7-fold increase, and significant delamination and oil droplet aggregation could be seen (Figure 5I). These findings suggest that the delamination and emulsion breakage of the monolayer emulsion increased significantly with storage temperature. In contrast, the droplet size of the bilayer emulsion when stored at 25 °C for 30 days was 4410 nm, a mere 3.3-fold increase, and its dispersion was still relatively uniform (Figure 5I,L). Viscosity was an important factor affecting emulsion stability, with the high viscosity of SA likely contributing significantly to the stability of the bilayer emulsion. Therefore, the bilayer emulsion was found to have superior dispersion and stability to the monolayer emulsion.

**Figure 6:**
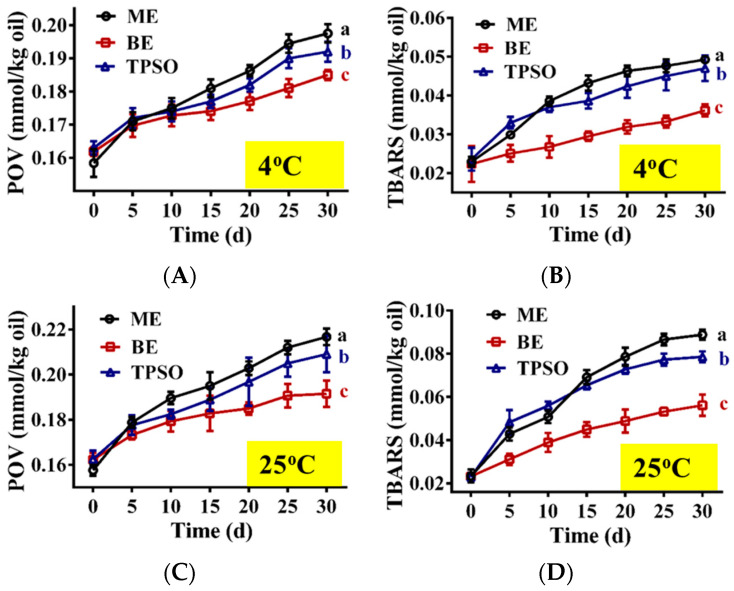
The oxidative stability of emulsions during storage (*n* = 3). (**A**,**B**) The POV (mmol/kg oil) and TBARS value (mmol/kg oil) at 4 °C, separately. (**C**,**D**) The POV (mmol/kg oil) and TBARS value (mmol/kg oil) at 25 °C. TPSO: tree peony seed oil; ME: monolayer emulsion; BE: bilayer emulsion. Two-way ANOVA followed by LSD test was used for statistical significance. Data marked with different superscript letters (a, b, c) differ significantly at *p* < 0.05.

The changes in the oxidation indexes of TPSO and the mono- and bilayer emulsions during storage are shown in Figure 6. At day 0, there was no significant difference in the POV and TBARS content of TPSO and the mono- and bilayer emulsions, suggesting that emulsification did not accelerate TPSO oxidation. As the storage time increased, varying degrees of oxidation were seen among the three samples, which was reflected in the increased POVs and TBARS content (Figure 6). The highest degree of oxidation was observed for the monolayer emulsion, followed by TPSO. The bilayer emulsion was the least oxidized. Both the POV and TBARS content of the monolayer emulsion were higher than those of TPSO alone due to the large surface area of the oils and fats in the emulsion. In addition, monolayer emulsions are less stable and prone to phase separation, and some TPSO was not fully encapsulated, thereby accelerating oxidation.

Compared to 4 °C (Figure 6A,B), storage at 25 °C significantly increased the degree of oxidation of TPSO and the mono- and bilayer emulsions (Figure 6C,D). The POV and TBARS content of the monolayer emulsion reached 0.217 and 0.089 mmol/kg oil, respectively, after 30 days of storage at 25 °C, an increase of 37.55% and 279.27%, respectively, from day 0. Under the same conditions, the POV and TBARS content of the bilayer emulsion were 0.192 and 0.056 mmol/kg oil, respectively, representing a 17.98% and 141.55% increase, respectively. This suggests that the bilayer emulsion significantly enhanced the oxidative stability compared to the monolayer emulsion. This is likely due to the thick and dense interfacial film formed by the electrostatic interactions between SA and WPI, separating TPSO from oxygen and other factors that accelerate oxidation. In addition, the CI of the monolayer emulsion increased with storage time, while that of the bilayer emulsion remained constant, with the CI of the bilayer emulsion being significantly smaller than that of the monolayer emulsion at both temperatures (Figure 5E,K). Therefore, layer-by-layer encapsulation of TPSO with WPI and SA was an effective strategy to prevent its oxidation.

### 3.8. Effect of pH on the Emulsion Stability

**Figure 7:**
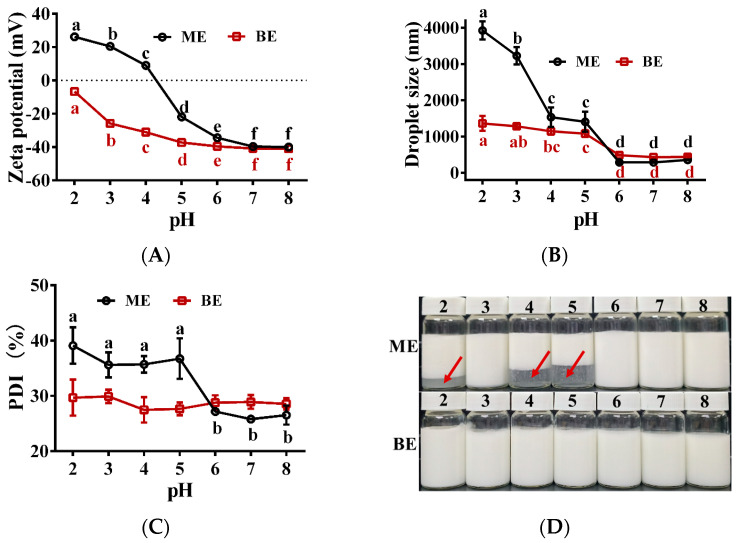
The effect of pH on the physical stability of emulsions (*n* = 3). (**A**–**D**) Zeta potential (mV), droplet size (nm), PDI (%) and the appearance of emulsion in two groups at different pH values. Red arrows represent significant delamination of the monolayer emulsion as the pH changes. ME: monolayer emulsion; BE: bilayer emulsion. One-way ANOVA followed by LSD test was used for statistical significance. Data marked with different superscript letters (a, b, c, d, e, f) differ significantly at *p* < 0.05.

The effect of different pH values on the stability of the emulsion is shown in Figure 7. At pH 6~8, the potential, droplet size, and PDI of both mono- and bilayer emulsions were relatively stable (Figure 7A–C). When pH < 6, these indicators of the monolayer emulsion increased as the pH decreased (Figure 7A–C). The monolayer emulsions also showed significant delamination (Figure 7D). These results indicate that monolayer emulsions were less stable in acidic environments. At pH < 6, the potential and droplet size of the bilayer emulsion also increased gradually with decreasing pH, but the increase was much smaller than that of the monolayer emulsion (Figure 7A,B). In addition, the bilayer emulsion did not show any delamination with pH change (Figure 7D). These results indicate that the bilayer emulsions were stable in pH 2~8 and therefore have good potential for application in acidic and weakly alkaline environments.

### 3.9. Effect of Metal Ionic on the Emulsion Stability

**Figure 8:**
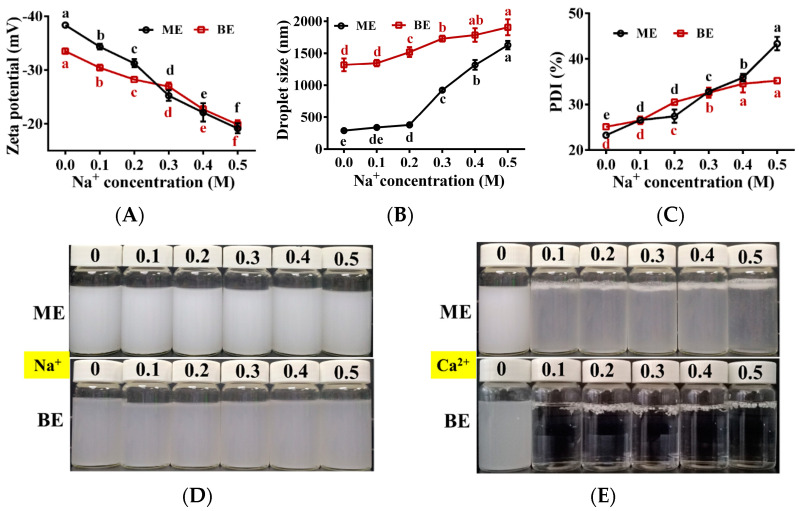
The effect of metal ion on the physical stability of emulsions (*n* = 3). (**A**–**E**) Zeta potential (mV), droplet size (nm), PDI (%) and the appearance of emulsion in two groups at different ionic strength. Na^+^: effect of sodium ion on the appearance of emulsion; Ca^2+^: effect of calcium ion on the appearance of emulsion. ME: monolayer emulsion; BE: bilayer emulsion. One-way ANOVA followed by LSD test was used for statistical significance. Data marked with different superscript letters (a, b, c, d, e, f) differ significantly at *p* < 0.05.

The effects of different concentrations of Na^+^ and Ca^2+^ on the stability of the emulsion are shown in Figure 8. With increasing Na^+^ concentration, the absolute value of zeta potential of mono-and bilayer emulsions kept decreasing, while the droplet size and PDI gradually increased (Figure 8A–C). This was due to the neutralization of Na^+^ with the negative charge on the droplet surface in the emulsion and the decrease of the net charge on the droplet surface, which weakened the electrostatic repulsion between the droplets [50]. Within these Na^+^ concentration ranges, both emulsions remained stable and did not delaminate (Figure 8D), indicating that both emulsions tolerated Na^+^ well. When the Na^+^ concentration increased from 0 to 0.5 M, the absolute values of zeta potential decreased by 50.1% and 40.7%, and the droplet size increased by 463.7% and 40.7% for the mono- and bilayer emulsions, respectively, indicating that the bilayer emulsion was more resistant to the potential and droplet size changes caused by Na^+^ and had better Na^+^ stability.

Flocculation and delamination were observed in monolayer emulsion in the presence of different concentrations of Ca^2+^ (0.1~0.5 M) (Figure 8E). This was due to the neutralization of Ca^2+^ with the negatively charged WPI, which weakened the electrostatic repulsive force between the droplets. Furthermore, the addition of Ca^2+^ changed the bilayer emulsion from a uniformly dispersed droplet to a gel state (Figure 8E), which was due to the formation of alginate beads by the ionic cross-linking reaction between Ca^2+^ and Na^+^ in sodium alginate [51]. These results demonstrate that mono- and bilayer emulsions were not well tolerated to Ca^2+^.

### 3.10. Gastric Digestion of TPSO Emulsions

**Figure 9:**
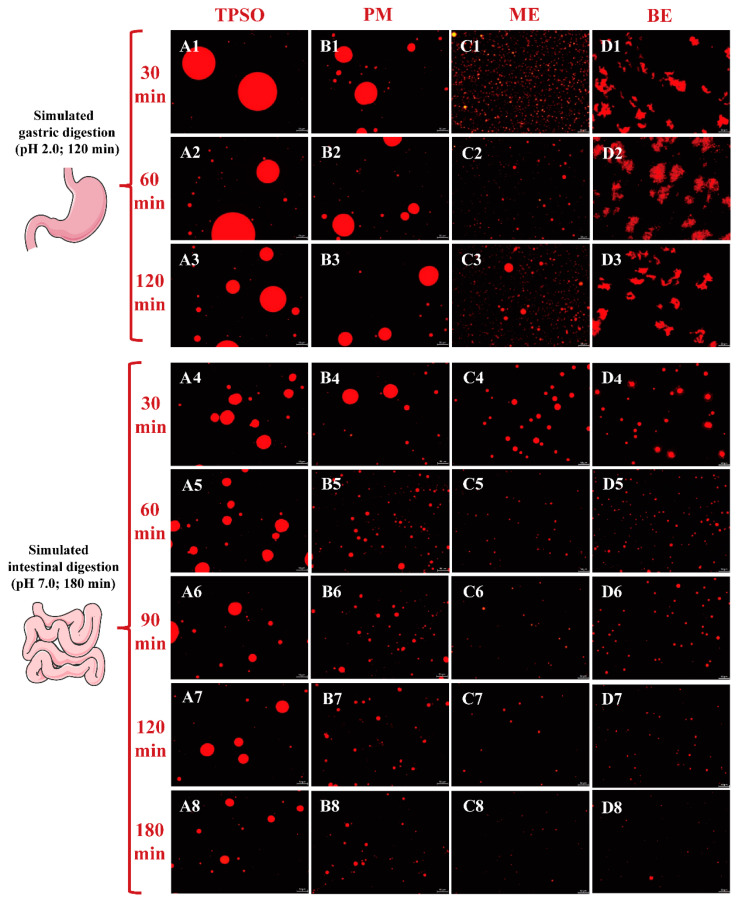
The microscopic images of different forms of TPSO in a simulated gastric fluid. TPSO (At: **A1**–**A8**, *t* = 1–8) tree peony seed oil; PM (Bt: **B1**–**B8**, *t* = 1–8) physical mixture; ME (Ct: **C1**–**C8**, *t* = 1–8) monolayer emulsion; BE (Dt: **D1**–**D8**, *t* = 1–8) bilayer emulsion. In addition, *t* = 1–3: the microscopic image of different forms of TPSO in simulated gastric fluid after 30, 60 and 120 min; *t* = 4–8: the microscopic image of different forms of TPSO in simulated intestinal fluid after 30, 60, 90, 120, and 180 min.

The microscopic images of different forms of TPSO in a simulated gastric fluid are shown in Figure 9. During gastric digestion, both TPSO and the physical mixture formed large oil droplets (Figure 9(A1–A3,B1–B3)). After 120 min of digestion, the oil droplets in the physical mixture were much smaller than in the TPSO sample (17,475 vs. 29,272 nm), suggesting that adding WPI resulted in a more uniform dispersion of the oil. This is likely because WPI can form an interfacial film at the oil–water interface during digestion, partially inhibiting the aggregation of oil droplets.

Both the monolayer emulsion and physical mixture contained the same amount of WPI and appeared to be similarly dispersed. However, the monolayer emulsion droplet size was much smaller than that of the physical mixture, and the droplets were more evenly distributed, potentially due to the high-pressure homogenization of the emulsion (Figure 9(C1–C3)). As digestion progressed in the stomach, the droplet size of the monolayer emulsion gradually increased due to the gradual hydrolysis of the WPI by pepsin, which broke down the interfacial film and exposed the oil droplets, allowing them to aggregate. However, the droplet size of the monolayer emulsion after 120 min digestion remained smaller than that of the physical mixture after only 30 min digestion, indicating that the monolayer emulsion had superior dispersion after gastric digestion.

The bilayer emulsion appeared in an aggregated state at all stages of gastric digestion (Figure 9(D1–D3)). This is because the bilayer emulsion remained negatively charged in the gastric environment (pH 2) while pepsin was positively charged, resulting in aggregation of the bilayer emulsion under the bridge effect of pepsin. Additionally, the positively charged pepsin could neutralize the negatively charged bilayer emulsion, resulting in a decrease in the absolute zeta potential of the emulsion and aggregation due to the reduced electrostatic repulsion.

### 3.11. Intestinal Digestive Properties

Following gastric digestion, the size and number of oil droplets in each group gradually decreased and became more uniformly distributed with increasing intestinal digestion time (0–180 min) (Figure 9(A4–D8)). This was due to the gradual hydrolysis of triglycerides by pancreatic lipase and the breakdown of the oil droplets. TPSO remained as large oil droplets throughout the digestion process, and the droplet size was reduced by 50% (180 min, 11,879 nm) compared to the initial size (0 min, 22,988 nm). The physical mixture group droplet size was reduced by more than 75%, likely because the WPI present in the physical mixture altered the oil–water interface and promoted triacylglycerol hydrolysis. Only a small number of small oil droplets were observed after 180 min of intestinal digestion of both the mono- and bilayer emulsions, likely because the smaller initial droplet size and larger specific surface area of both made them more easily hydrolyzed by pancreatic lipase. Previous studies have also found that emulsion can be used as a delivery system to promote the improvement of bioavailability [52,53,54]. The difference in droplet size between the mono- and bilayer emulsions prior to digestion was significant, but the droplets were comparable in size after 180 min of intestinal digestion. This result agrees with a study by Li et al. (2016) [55]. However, previous studies have reported that the lipid digestibility of a cod liver oil emulsion gradually decreased with increasing numbers of emulsion interfacial layers [56]. However, this finding may have been related to the use of different emulsifiers and wall materials rather than the absolute number of layers. In addition, the bilayer emulsion significantly aggregated in the simulated gastric fluid but was uniformly dispersed in the simulated intestinal fluid, likely due to environmental differences such as pH. These results indicate that ME and BE were more easily digested and absorbed than TPSO and PM.

### 3.12. FFA Release and ALA Bioaccessibility

**Figure 10:**
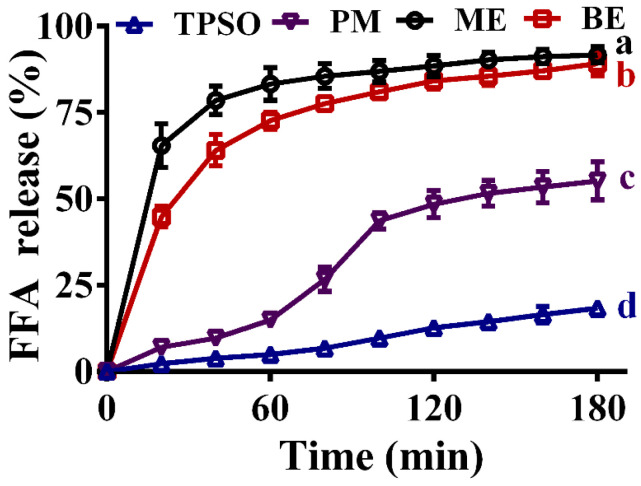
Fatty acid release rates of TPSO and their emulsions in the simulated intestinal fluid. TPSO: tree peony seed oil (*n* = 3); PM: physical mixture (*n* = 3); ME: monolayer emulsion (*n* = 3); BE: bilayer emulsion (*n* = 3). Two-way ANOVA followed by LSD test was used for statistical significance. Groups with different superscript letters (a, b, c, d) were significantly different at *p* < 0.05.

The fatty acid release rates of TPSO and the mono- and bilayer emulsions in the simulated intestinal fluid are shown in Figure 10. Of the four samples, TPSO released the least FFA and had the slowest release rate, with less than 20% of FFA released after 180 min of digestion in the simulated intestinal fluid, confirming the low bioavailability of TPSO. In the physical mixture group, the addition of WPI resulted in a significant increase in both the amount of FFA released and the release rate, with nearly 50% of FFA released after 180 min of digestion. For both samples, the TPSO existed as larger oil droplets, making it difficult to emulsify the oil adequately in the absence of an emulsifier. As a result, TPSO could float on the surface of the simulated digestion solution and was not in complete contact with the digestive solution, which directly determined the low decomposition rate.

The FFA release rate from the mono- and bilayer emulsions was highest in the first 20 min, and then gradually stabilized. At the end of intestinal digestion (180 min), the amounts of FFA released from the mono- and bilayer emulsions were comparable, with the vast majority being digested (93.3% vs. 89.2%, respectively). This indicates that the polymeric electrolyte layer did not hinder the digestion and absorption of TPSO by the intestine. A similar pattern of FFA release was reported by Sun et al. (2022) [57]. Both the rate and amount of FFA release were significantly enhanced in the mono- and bilayer emulsion groups compared to the TPSO and physical mixture groups, indicating that the formation of TPSO emulsions can accelerate its digestion and absorption. While monolayer emulsion exhibited similar intestinal digestive properties and fatty acid release rates to bilayer emulsion, the latter showed significant advantages in terms of physical stability, oxidative stability, and environmental stability. As a result, bilayer emulsions have more potential for application in the efficient delivery of TPSO.

**Figure 11:**
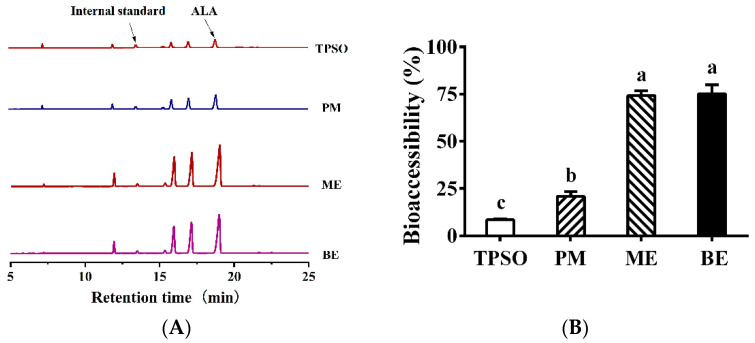
Fatty acid profile in micelles and the bioaccessibility of ALA in the emulsion. (**A**) Fatty acid profile in micelles in TPSO, PM, ME, and BE; (**B**) The bioaccessibility of ALA in TPSO, PM, ME, and BE; TPSO: tree peony seed oil (*n* = 3); PM: physical mixture (*n* = 3); ME: monolayer emulsion (*n* = 3); BE: bilayer emulsion (*n* = 3). Two-way ANOVA followed by LSD test was used for statistical significance. Data marked with different superscript letters (a, b, c) differ significantly at *p* < 0.05.

ALA is a major fatty acid in TPSO. In Figure 11A, the peak of ALA was the largest in the mono- and bilayer emulsions, followed by PM, while TPSO was the lowest. This indicates that the fatty acid content of micelles increased when TPSO was present in the form of emulsions. In Figure 11B, the bioaccessibility of ALA was less than 30% in the TPSO and PM groups, while it was 75.1% and 74.2% in the ME and BE groups, respectively. Compared to TPSO, the bioaccessibility of mono- and bilayer emulsions increased by 792% and 781%, respectively, indicating that the presence of TPSO in the form affects the absorption of ALA. Such results were consistent with a 180-min FFA release from intestinal digestion (Figure 10). This may be due to the fact that emulsions have a larger surface area, providing more lipase binding sites and increasing lipid hydrolysis, thus improving the bioaccessibility of ALA [58]. Similarly, Gayoso et al. (2018) found that the bioaccessibility of DHA was significantly enhanced when algal oil was delivered using gelled emulsions compared to non-emulsified oils [30].

TPSO is a novel woody oil rich in n-3 PUFA that is gaining increasing attention for its excellent biological activities such as antioxidant. In this study, the TPSO bilayer emulsions were prepared using electrostatic layer self-assembly technique to improve its stability and bioaccessibility. The results showed that the bilayer emulsions with WPI and SA as the emulsifier and polyelectrolyte, respectively, significantly improved the oxidative stability of TPSO, as evidenced by the significant reduction in POV and TBARS at 4 °C and 25 °C. Similarly, the physical stability of the bilayer emulsions during storage was significantly improved compared to the monolayer emulsions as evidenced by the smaller droplet size, PDI, CI, microscopic image, and appearance at 25 °C. In addition, this bilayer emulsions showed stable tolerance to pH and sodium ions. These results consistently indicate that TPSO bilayer emulsions significantly improve the environmental stability and physical and oxidative stability of TPSO during storage compared to monolayer emulsions and physical blends. In addition, in vitro simulated digestion experiments showed that digestion of this bilayer emulsion occurred mainly in the intestinal phase, with significantly higher fatty acid release rates and bioaccessibility of ALA than the physical blend group. Based on these results, the TPSO bilayer emulsion prepared by the electrostatic layer self-assembly method significantly improved the stability and bioaccessibility of TPSO.

## 4. Conclusions

In this study, a bilayer emulsion containing WPI and SA produced using an electrostatic layer-by-layer self-assembly technique was an effective delivery strategy for TPSO. This bilayer emulsion showed significantly improved oxidative stability and other physicochemical properties (rheological properties and physical and environmental stability). Besides, such bilayer emulsion was more easily digested and absorbed than TPSO, showing faster FFA release rates and higher ALA bioaccessibility. This novel strategy contributes to the development of TPSO as a functional food.

## Figures and Tables

**Table 1:** The secondary structure of WPI in mono- and bilayer emulsions.

	α-Helix	β-Sheet	β-Turn	Random Coil
WPI	28.73 ± 1.02 a	17.90 ± 0.20 b	19.63 ± 0.59 c	35.27 ± 1.46 a
ME	13.75 ± 1.63 c	29.90 ± 1.84 a	21.05 ± 0.49 b	36.00 ± 0.42 a
BE	17.27 ± 0.76 b	28.57 ± 0.65 a	24.47 ± 0.35 a	31.57 ± 0.55 b

WPI: whey protein isolate; ME: monolayer emulsion; BE: bilayer emulsion; Data marked with different superscript letters (a, b, c) differ significantly at *p* < 0.05 on a column.

## Data Availability

Not applicable.

## Supplementary Materials

The following supporting information can be downloaded at: https://www.mdpi.com/article/10.3390/antiox12051128/s1, Figure S1: Physical properties, microscopic image, and appearance of monolayer emulsion prepared with different concentrations of WPI; Figure S2: Physical properties, microscopic image, and appearance of TPSO bilayer emulsions produced using different concentrations of SA; Table S1: Composition of major fatty acids and beneficial components in tree peony seed oil.

## References

[1] Liu P., Zhang L., Wang X., Gao J., Yi J., Deng R. (2019). Characterization of Paeonia ostii seed and oil sourced from different cultivation areas in China. Ind. Crops Prod..

[2] Chang M., Wang Z., Zhang T., Wang T., Liu R., Wang Y., Jin Q., Wang X. (2020). Characterization of fatty acids, triacylglycerols, phytosterols and tocopherols in peony seed oil from five different major areas in China. Food Res. Int..

[3] Wang X., Li C., Contreras M.M., Verardo V., Gómez-Caravaca A.M., Xing C. (2020). Integrated Profiling of Fatty Acids, Sterols and Phenolic Compounds in Tree and Herbaceous Peony Seed Oils: Marker Screening for New Resources of Vegetable Oil. Foods.

[4] Mao Y., Han J., Tian F., Tang X., Hu Y., Guan Y. (2017). Chemical Composition Analysis, Sensory, and Feasibility Study of Tree Peony Seed. J. Food Sci..

[5] Kwek E., Zhu H., Ding H., He Z., Hao W., Liu J., Ma K., Chen Z. (2022). Peony seed oil decreases plasma cholesterol and favorably modulates gut microbiota in hypercholesterolemic hamsters. Eur. J. Nutr..

[6] Yang X., Zhang D., Song L.M., Xu Q., Li H., Xu H. (2017). Chemical profile and antioxidant activity of the oil from peony seeds (*Paeonia suffruticosa* Andr.). Oxidative Med. Cell. Longev..

[7] He W.S., Rui J., Wang Q., Chen Z. (2021). Antioxidant Activity of Piceatannol in Canola Oil. Eur. J. Lipid Sci. Technol..

[8] Couëdelo L., Boué-Vaysse C., Fonseca L., Montesinos E., Djoukitch S., Combe N., Cansell M. (2011). Lymphatic absorption of α-linolenic acid in rats fed flaxseed oil-based emulsion. Brit. J. Nutr..

[9] Sugasini D., Lokesh B.R. (2012). Uptake of α-linolenic acid and its conversion to long chain omega-3 fatty acids in rats fed microemulsions of linseed oil. Lipids.

[10] Cong L., Wang J., Lu H., Tian M., Ying R., Huang M. (2023). Influence of different anionic polysaccharide coating on the properties and delivery performance of nanoliposomes for quercetin. Food Chem..

[11] Chen P., Yang B.Q., Wang R.M., Xu B.C., Zhang B. (2022). Regulate the interfacial characteristic of emulsions by casein/butyrylated dextrin nanoparticles and chitosan based on ultrasound-assisted homogenization: Fabrication and characterization. Food Hydrocoll..

[12] Hao H., Jin X., Liu Y. (2021). Study on preparation and stability of peony seed oil nanoemulsion by ultra high pressure homogenization. Cereals Oils.

[13] Yang C., Han R., Kong F., Lei F., He D., Luo Z. (2021). Preparation and properties of peony seed oil microemulsion. China Oils Fats.

[14] Gan C., Liu Q., Zhang Y., Shi T., He W.S., Jia C. (2022). A novel phytosterols delivery system based on sodium caseinate-pectin soluble complexes: Improving stability and bioaccessibility. Food Hydrocoll..

[15] Li M., Sun Y., McClements D.J., Yao X., Ma C., Liu X., Liu F. (2022). Interfacial engineering approaches to improve emulsion performance: Properties of oil droplets coated by mixed, multilayer, or conjugated lactoferrin-hyaluronic acid interfaces. Food Hydrocoll..

[16] Martel-Estrada S.A., Morales-Cardona A.I., Vargas-Requena C.L., Rubio-Lara J.A., Martínez-Pérez C.A., Jimenez-Vega F. (2022). Delivery systems in nanocosmeceuticals. Rev. Adv. Mater. Sci..

[17] Zhu Y., Fu S., Wu C., Qi B., Teng F., Wang Z., Li Y., Jiang L. (2020). The investigation of protein flexibility of various soybean cultivars in relation to physicochemical and conformational properties. Food Hydrocoll..

[18] Wang Y., Zheng Z., Wang K., Tang C., Liu Y., Li J. (2020). Prebiotic carbohydrates: Effect on physicochemical stability and solubility of algal oil nanoparticles. Carbohyd. Polym..

[19] Liu Y., Zhang W., Wang K., Bao Y., Regenstein J.M., Zhou P. (2019). Fabrication of gel-like emulsions with whey protein isolate using microfluidization: Rheological properties and 3D printing performance. Food Bioprocess Technol..

[20] Li Y., Xiang D. (2019). Stability of oil-in-water emulsions performed by ultrasound power or high-pressure homogenization. PLoS ONE.

[21] Hou Z., Zhang M., Liu B., Yan Q., Yuan F., Xu D., Gao Y. (2012). Effect of chitosan molecular weight on the stability and rheological properties of β-carotene emulsions stabilized by soybean soluble polysaccharides. Food Hydrocoll..

[22] Lv Y., Zhang X., Abbas S., Karangwa E. (2012). Simplified optimization for microcapsule preparation by complex coacervation based on the correlation between coacervates and the corresponding microcapsule. J. Food Eng..

[23] Javier L.V., Ricardo V.C., Giovanna F., Francesco D., Rommy N.Z., Carolina S., Tatiana B.I. (2020). Influence of interfacial structure on physical stability and antioxidant activity of curcumin multilayer emulsions. Food Bioprod. Process..

[24] Gudipati V., Sandra S., McClements D.J., Decker E.A. (2010). Oxidative stability and in vitro digestibility of fish oil-in-water emulsions containing multilayered membranes. J. Agric. Food Chem..

[25] Chen S., Zhang N., Tang C.H. (2016). Influence of nanocomplexation with curcumin on emulsifying properties and emulsion oxidative stability of soy protein isolate at pH 3.0 and 7.0. Food Hydrocoll..

[26] Ding J., Xu Z., Qi B., Cui S., Wang T., Jiang L., Zhang Y., Sui X. (2019). Fabrication and characterization of soybean oil bodies encapsulated in maltodextrin and chitosan-EGCG conjugates: An in vitro digestibility study. Food Hydrocoll..

[27] Chen E., Wu S., McClements D.J., Li B., Li Y. (2017). Influence of pH and cinnamaldehyde on the physical stability and lipolysis of whey protein isolate-stabilized emulsions. Food Hydrocoll..

[28] Minekus M., Alminger M., Alvito P., Balance S., Bohn T., Bourlieu C., Carrière F., Boutrou R., Corredig M., Dupont D. (2014). A standardised static in vitro digestion method suitable for food—An international consensus. Food Funct..

[29] Gasa-Falcon A., Acevedo-Fani A., Oms-Oliu G., Odriozola-Serrano I., Martín-Belloso O. (2020). Development, physical stability and bioaccessibility of β-carotene-enriched tertiary emulsions. J. Funct. Foods.

[30] Gayoso L., Ansorena D., Astiasarán I. (2018). DHA rich algae oil delivered by O/W or gelled emulsions: Strategies to increase its bioaccessibility. J. Sci. Food Agric..

[31] He W.S., Sun Y., Li Z., Yang H., Li J., Wang Q., Tan C., Zou B. (2022). Enhanced antioxidant capacity of lipoic acid in different food systems through lipase-mediated esterification with phytosterols. J. Sci. Food Agric..

[32] He W.S., Li L., Rui J., Li J., Sun Y., Cui D., Xu B. (2020). Tomato seed oil attenuates hyperlipidemia and modulates gut microbiota in C57BL/6J mice. Food Funct..

[33] Boye J.I., Aksay S., Roufik S., Ribéreau S., Mondor M., Farnworth E., Rajamohamed S.H. (2009). Comparison of the functional properties of pea, chickpea and lentil protein concentrates processed using ultrafiltration and isoelectric precipitation techniques. Food Res. Int..

[34] Midekessa G., Godakumara K., Ord J., Viil J., Lättekivi F., Dissanayake K., Kopanchuk S., Rinken A., Andronowska A., Bhattacharjee S. (2020). Zeta potential of extracellular vesicles: Toward understanding the attributes that determine colloidal stability. ACS Omega.

[35] Prakash A., Baskaran R., Paramasivam N., Vadivel V. (2018). Essential oil based nanoemulsions to improve the microbial quality of minimally processed fruits and vegetables: A review. Food Res. Int..

[36] Niu F., Niu D., Zhang H., Chang C., Gu L., Su Y., Yang Y. (2016). Ovalbumin/gum arabic-stabilized emulsion: Rheology, emulsion characteristics, and Raman spectroscopic study. Food Hydrocoll..

[37] Smułek W., Siejak P., Fathordoobady F., Masewicz Ł., Guo Y., Jarzębska M., Kitts D.D., Kowalczewski P.Ł., Baranowska H.M., Stangierski J. (2021). Whey Proteins as a Potential Co-Surfactant with *Aesculus hippocastanum* L. as a Stabilizer in Nanoemulsions Derived from Hempseed Oil. Molecules.

[38] Sun J., Liu W., Feng M., Xu X., Zhu G. (2019). Characterization of olive oil emulsions stabilized by flaxseed gum. J. Food Eng..

[39] Chen M., Xu F., Nsor-Atindana J., Chen X., Liu F., Wu J., Zhong F. (2022). High protein and high oil emulsions: Phase diagram, stability and interfacial adsorption. LWT.

[40] Li S., Sun J., Yan J., Zhang S., Shi C., McClements D.J., Liu X., Liu F. (2020). Development of antibacterial nanoemulsions incorporating thyme oil: Layer-by-layer self-assembly of whey protein isolate and chitosan hydrochloride. Food Chem..

[41] Su J., Guo Q., Cai Y., Wang T., Mao L., Gao Y., Yuan F., der Meeren P.V. (2020). Effect of Ultra-high temperature processing on the physicochemical properties and antibacterial activity of d -limonene emulsions stabilized by β-lactoglobulin/Gum arabic bilayer membranes. Food Chem..

[42] Liao Y., Sun Y., Peng X., Wang Q., Wu L., Yan S., Liu G., Zhu C., Qi B., Li Y. (2022). Preparation of perilla oil multilayer emulsion and the in vitro digestibility of emulsion oil. Food Sci..

[43] Liu L., Zhao Q., Liu T., Kong J., Long Z., Zhao M. (2012). Sodium caseinate/carboxymethylcellulose interactions at oil–water interface: Relationship to emulsion stability. Food Chem..

[44] Sabet S., Seal C.K., Swedlund P.J., McGillivray D.J. (2020). Depositing alginate on the surface of bilayer emulsions. Food Hydrocoll..

[45] Zhang C., Cai Y., Peng C., Wang Z. (2022). Preparation and stability of DHA algae oil nanoemulsion. J. Chin. Cereals Oils Assoc..

[46] Wang H., Zhang R., Wu M., Yang N., Ye S., Shuai X., Jiang S., Li Y., He J. (2022). Preparation and characterization of bitter gourd seed oil multilayer emulsion based on the electrostatic layer-by-Layer self-assembly technique. Sci. Technol. Food Ind..

[47] Lee S.H., Lefèvre T., Subirade M., Paquin P. (2008). Effects of ultra-high pressure homogenization on the properties and structure of interfacial protein layer in whey protein-stabilized emulsion. Food Chem..

[48] Han M., Wu Y., Wang P., Xu X., Zhou G. (2015). The changes and relationship of structure and functional properties of rabbit myosin during heat-induced gelation. CyTA—J. Food.

[49] Sun C., Liang B., Sheng H., Wang R., Zhao J., Zhang Z., Zhang M. (2018). Influence of initial protein structures and xanthan gum on the oxidative stability of O/W emulsions stabilized by whey protein. Int. J. Biol. Macromol..

[50] Chen W., Wang W., Guo M., Li Y., Meng F., Liu D. (2022). Whey protein isolate-gum Acacia Maillard conjugates as emulsifiers for nutraceutical emulsions: Impact of glycation methods on physicochemical stability and in vitro bioaccessibility of β-carotene emulsions. Food Chem..

[51] Zhang Y., Yang Y., Zhao X., Gao J. (2023). Investigation on ionical cross-linking of alginate by monovalent cations to fabrication alginate gel for biomedical application. React. Funct. Polym..

[52] Zhang Y., Yang Y., Mao Y., Zhao Y., Li X., Hu J., Li Y. (2022). Effects of mono- and di-glycerides/phospholipids (MDG/PL) on the bioaccessibility of lipophilic nutrients in a protein-based emulsion system. Food Funct..

[53] Gao Y., Yuan S., Chen Y., Liu F., Wei Z., Cao W., Li R.W., Xu J., Xue C., Tang Q. (2022). The improvement effect of astaxanthin-loaded emulsions on obesity is better than that of astaxanthin in the oil phase. Food Funct..

[54] Li R., Yuan G., Li D., Xu C., Du M., Tan S., Liu Z., He Q., Rong L., Li J. (2022). Enhancing the bioaccessibility of puerarin through the collaboration of high internal phase Pickering emulsions with β-carotene. Food Funct..

[55] Li J., Lin C., Liu Y. (2016). Preparation and stability of multilayer emulsions of linseed oil by electrostatic layer-by-layer deposition. Food Sci..

[56] Boonlao N., Shrestha S., Sadiq M.B., Anal A.K. (2020). Influence of whey protein-xanthan gum stabilized emulsion on stability and in vitro digestibility of encapsulated astaxanthin. J. Food Eng..

[57] Sun M., Quan S., Chen Y., Chen H., Peng D., Deng Q. (2022). Effect of calcium ions on storage properties and astaxanthin delivery efficiency of DHA algae oil-loaded emulsion using flaxseed gum-perilla protein isolate. China Oils Fats.

[58] Hu M., Xie F., Zhang S., Qi B., Li Y. (2020). Effect of nanoemulsion particle size on the bioavailability and bioactivity of perilla oil in rats. J. Food Sci..

