# Supplementary material for: Enhancing the Stability and Bioaccessibility of Tree Peony Seed Oil Using Layer-by-Layer Self-Assembling Bilayer Emulsions
Source: Antioxidants (Basel). 2023 May 20;12(5):1128. doi: 10.3390/antiox12051128 (PMC10215233; doi:10.3390/antiox12051128)

Enhancing the stability and bioaccessibility of tree peony seed oil using layer-by-layer self-assembling bilayer emulsions

Wen-Sen He,[a,*] Qingzhi Wang,[a] Zhishuo Li,[a] Jie Li,[a] Liying Zhao,[a] Junjie Li,[a] Chen Tan,[b] Fayong Gong [c]

[a] *School of Food and Biological Engineering, Jiangsu University, 301 Xuefu Road, Zhenjiang 212013, Jiangsu, China*

[b] *Beijing Engineering and Technology Research Center of Food Additives, Beijing Technology & Business University (BTBU), Beijing 100048, China*

[c] *Panxi Crops Research and Utilization Key Laboratory of Sichuan Province*, *Xichang University*, *Xichang* 615013, *China*

*Corresponding Author.

E-mail: wshe2013@163.com (HE W. S.)

Table S1 Composition of major fatty acids and beneficial components in tree peony seed oil.

	Compositions	Content
Fatty acid (mg/g)	C16:0	42.87±3.32
	C18:0	8.39±0.67
	C18:1	169.97±12.36
	C18:2	192.59±14.57
	C18:3	301.15±22.22
Beneficial components	Total phenols (mg GAE/kg)	17.22±0.47
	Total flavonoids (mg RAE/kg)	22.33±1.85
	γ-Tocopherol (mg/100g)	13.87±1.33
	Squalene (mg/100g)	2.58±0.30
	β-Sitosterol (mg/100g)	182.94±12.90

Figure S1 Physical properties, microscopic images, and appearance of monolayer emulsion prepared with different concentrations of WPI. (A) Zeta potential (mV). (B) Particle size (nm). (C) PDI (%). (D) Encapsulation efficiency (%). (E)-(I) The appearance and microscopic image of emulsion in 0.5-2.5% of WPI. One-way ANOVA followed by LSD test was used for statistical significance. Data are expressed as the means with different superscript letters (a, b, c) differ significantly at $p < 0.05$.

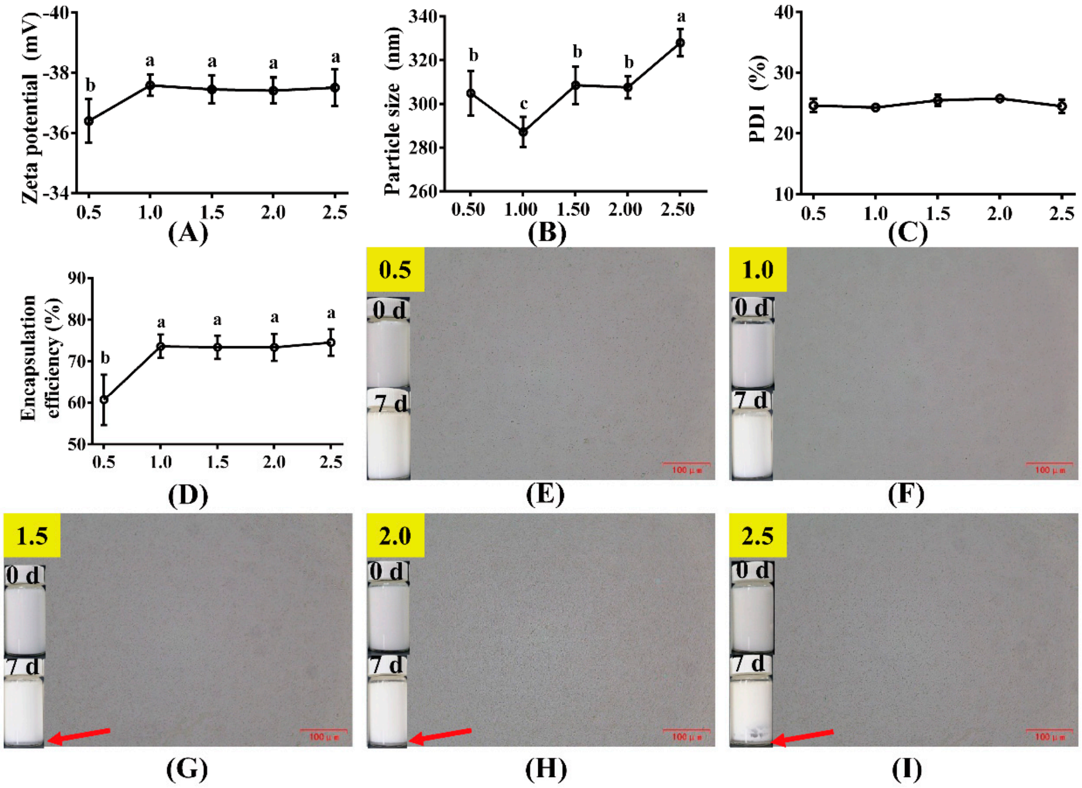

Figure S2 Physical properties, microscopic images and appearance of TPSO bilayer emulsions produced using different concentrations of SA. (A) Zeta potential (mV). (B) Particle size (nm). (C) PDI (%). (D) Encapsulation efficiency (%). (E)-(J) The microscopic images of emulsion in 0.25-1.5% of SA group. (K) and (L) The appearance of emulsion in 0.25-1.5% of SA group after 0 and 7 days. One-way ANOVA followed by LSD test was used for statistical significance. Data are expressed as the means with different superscript letters (a, b, c) differ significantly at $p < 0.05$.

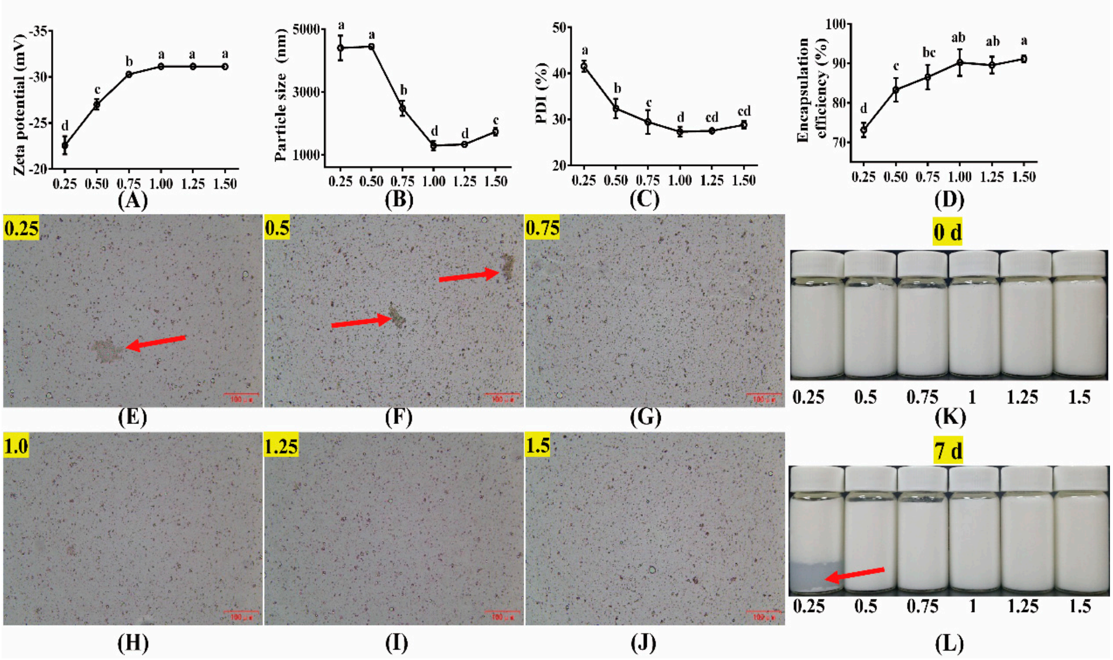

Supplement: Supplementary file 1 [file antioxidants-12-01128-s001.zip › antioxidants-2379257-supplementary.pdf]
